# Comparison of various chemometric analysis for rapid prediction of thiobarbituric acid reactive substances in rainbow trout fillets by hyperspectral imaging technique

**DOI:** 10.1002/fsn3.1043

**Published:** 2019-04-24

**Authors:** Sara Khoshnoudi‐Nia, Marzieh Moosavi‐Nasab

**Affiliations:** ^1^ Seafood Processing Research Group & Department of Food Science and Technology, School of Agriculture Shiraz University Shiraz Iran

**Keywords:** chemometric analysis, linear regression, lipid oxidation, malondialdehyde, nonlinear regression, rainbow trout fish

## Abstract

This study explores the potential application of hyperspectral imaging (HSI; 430–1,010 nm) coupled with different linear and nonlinear models for rapid nondestructive evaluation of thiobarbituric acid‐reactive substances (TBARS) value in rainbow trout (*Oncorhynchus mykiss)* fillets during 12 days of cold storage (4 ± 2°C). HSI data and TBARS value of fillets were obtained in the laboratory. The primary prediction models were established based on linear partial least squares regression (PLSR) and least squares support vector machine (LS‐SVM). In full spectral range, the prediction capability of LS‐SVM ($R_{\text{P}}^{2}$ = 0.829; RMSEP = 0.128 mg malondialdehyde [MDA]/kg) was better than PLSR ($R_{\text{P}}^{2}$ = 0.748; RMSEP = 0.155 mg MDA/kg) model and LS‐SVM model exhibited satisfactory prediction performance ($R_{\text{P}}^{2}$ > 0.82). To simplify the calibration models, a combination of uninformative variable elimination and backward regression (UB) was used as variable selection. Nine wavelengths were selected. Various chemometric analysis methods including linear PLSR and multiple linear regression and nonlinear LS‐SVM and back‐propagation artificial neural network (BP‐ANN) were compared. The simplified models showed better capability than those were built based on the whole dataset in prediction of TBARS values. Moreover, the nonlinear models were preferred over linear models. Among the four chemometric algorithms, the best and weakest models were LS‐SVM and PLSR model, respectively. UB‐LS‐SVM model was the optimal models for predicting TBARS value in rainbow trout fillets ($R_{\text{P}}^{2}$ = 0.831; RMSEP = 0.125 mg MDA/kg). The establishing of lipid‐oxidation prediction model in rainbow trout fish was complicated, due to the fluctuations of TBARS values during storage. Therefore, further researches are needed to improve the prediction results and applicability of HIS technique for prediction of TBARS value in rainbow trout fish.

## 1 INTRODUCTION

Fresh fish is a very perishable food due to various reasons such as large amounts of unsaturated fatty acids, high content of free amino acids and volatile nitrogen bases, and consequently high final pH (Ashie, Smith, Simpson, & Haard, 1996; Barkhori‐Mehni, Khanzadi, Hashemi, & Azizzadeh, 2017). Rainbow trout (*Oncorhynchus mykiss*) is a kind of popular aquaculture species in the world which is recognized as a fatty fish species (Rezaei & Hosseini, 2008; Venugopal & Shahidi, 1996). Therefore, it is highly susceptible to lipid oxidation during storage (Xu, Riccioli, & Sun, 2016). Lipid oxidation is a major quality problem resulting in oxidative rancidity (off‐odors and off‐flavor) in fatty foods (Rezaei & Hosseini, 2008; Sedaghat, Mohammad Hosseini, Khoshnoudi‐nia, Najafi, & Koocheki, 2015). Thiobarbituric acid‐reactive substances (TBARS) are the most commonly used indicator for monitoring overall lipid peroxidation (Nichi et al., 2006).

Thiobarbituric acid‐reactive substances test is based on spectrophotometric measurement of the colored malondialdehyde (MDA)–TBA complex evaluating amount of MDA in samples. Although TBARS test shows relatively exact result, this method is time‐, labor‐, and chemical solvent‐consuming and destructive. Hyperspectral imaging (HSI) techniqueby integrating the advantages of two important nondestructive methods, spectroscopy and computer vision, into one system has become a promising tool to determine and evaluate food quality and safety in a nondestructive and rapid manner (ElMasry, Barbin, Sun, & Allen, 2012; Kamal & Karoui, 2015; Naganathan et al., 2008; Pu, Liu, Wang, & Sun, 2016; Wang et al., 2015; Wu & Sun, 2013a, 2013b; Xiong, Sun, Xie, Han, & Wang, 2015). In this regard, several exploratory studies were established about using HSI coupled with the appropriate linear and/or nonlinear chemometric multivariate analyses to evaluate quality and safety of fish and other seafood based on various important parameters such as sensory parameters (Cheng & Sun, 2015a; Ma, Sun, Qu, & Pu, 2017; Wu, Sun, & He, 2012), TVB‐N (Cheng, Sun, & Wei, 2017; Cheng, Sun, Zeng, & Pu, 2014), TVC (Cheng & Sun, 2015b; Khoshnoudi‐Nia, Moosavi‐Nasab, Nassiri, & Azimifar, 2018; Wu & Sun, 2013c), *K*‐value (Cheng, Sun, Pu, & Zhu, 2015; Cheng et al., 2016), and TBARS (Cheng, Sun, Pu, Wang, & Chen, 2015; Cheng et al., 2016; Xu et al., 2016). The results of these researches have shown the good potential of HSI system for quality assessment of seafood. However, to the best of our knowledge, up to now, no investigation on determination of TBARS value in rainbow trout fillet using HSI technique has been reported. Moreover, previous studies investigating the application of HSI technique for rapid determination of TBARS value in grass carp and salmon fillets were based on linear calibration model. To improve the prediction performance of TBARS values, more analysis strategies should be investigated and compared. Therefore, the purpose of this study was to (a) investigate the suitability of using HSI (400–1,000 nm) for nondestructive evaluation of TBARS value in rainbow trout fish fillets; (b) compare the prediction performance of four chemometric methods (linear: partial least squares regression [PLSR] and multiple linear regression [MLR]; nonlinear: back‐propagation artificial neural network [BP‐ANN] and least squares support vector machine [LS‐SVM] models); (c) select the most informative wavelengths linked to TBARS prediction by a novel combination of uninformative variable elimination (UVE) and backward regression (UB), and (d) development the simplified model with potential of on/inline evaluation of TBARS value in rainbow trout fillets.

## 2 MATERIALS AND METHODS

### 2.1 Sample preparation and storage condition

Forty aquaculture rainbow trout (average weight and length 700–1200 g and 35–45 cm, respectively) were purchased from a local aquaculture farm located at Baja (Shiraz, Fars, Iran). Fish samples were harvested in December 2017 and slaughtered by keeping away from water. After that, whole rainbow trout fishes immediately transported to the Seafood Processing Research Group laboratory, Shiraz University, Iran, in insulated ice boxes. After rigor mortis was passed, the fish samples were beheaded, gutted, filleted, and washed with cold water. Fish fillets were cut into a rectangular shape (length × width ×thickness: 8.0 × 4.0 × 1.0 cm), and 147 subsamples were obtained (calibration set: 98 subsamples and prediction set: 49 samples). Fillets were randomly divided into seven groups, labeled, packaged into plastic bags, and subjected to cold storage (4 ± 2°C) for 0, 2, 4, 6, 8, 10, and 12 days.

### 2.2 Hyperspectral image system

A HSI system in the reflectance mode was assembled to acquire hyperspectral images of fish fillets. The system consisted of an imaging spectrograph (Hyper Spectral Imaging 1000; Opt Co.), an illumination system, a mobile platform, and computer control system. This system covered the wavelength range of 400–1,160 nm. The detailed description of the system is available in Khoshnoudi‐Nia et al. (2018) manuscript.

### 2.3 Image acquisition and calibration

For every cold storage period, each of the subsamples was placed on the mobile platform and scanned by the HSI system. When the sample was scanned, a corresponding three‐dimensional image (hypercube) with one spectral dimension (*z*) and two spatial dimensions (*x*, *y*) was acquired storing in a raw format. Besides, the CCD detector of HSI systems computed the raw data with signal intensity (not with spectral reflectance). Therefore, the raw images should be calibrated by images of white Teflon tile (99% reflectance) and the dark image (0% reflectance) to eliminate the impacts of the illumination (Wu & Sun, 2013a).

### 2.4 Measurement TBA values

For each cold storage period, subsamples were first scanned by the HSI system and then TBARS values of them were measured using traditional methods describing by Sun, Faustman, Senecal, Wilkinson, and Furr (2001) and presented as mg MDA/kg (Sun et al., 2001).

### 2.5 Regions of interest identification and average spectra extraction

After the acquisition and calibration of hyperspectral images, the regions of interest (ROIs) with rectangle shapes were selected. The average spectra of all pixels within each region were extracted by ROI tool in ENVI v5.4 software (ITT Visual Information Solutions; Research Systems Inc.) (Cheng, Sun, Pu, Wang, et al., 2015).

### 2.6 Spectral preprocessing

In order to align the undesired noises of the extracted average spectra and better predicting performance, Savitzky–Golay (S‐G) technique was applied as preprocessing of spectral data. The S‐G operation was carried out by Unscrambler software (version 10.4; CAMO).

### 2.7 Chemometric modeling

In this study, PLSR and MLR models as the most widely applied and robust linear regression methods were used to establish calibration models between the spectral data of fish fillets samples and their reference TBARS content. However, the spectra extraction from HSI technology may be polluted by some uncertain and nonlinear parameters such as stray light (Zhang et al., 2018). Therefore, LS‐SVM and BP‐ANN methods as nonlinear calibration models were also established for comparison. Establishing PLSR and MLR models were carried out in Unscrambler 10.4x software (CAMO), and LS‐SVM and BP‐ANN models were built in MATLAB R2016a (The Mathworks Inc.).

### 2.8 Important wavelength selection

Hyperspectral image systems consisted of a large degree of spectral data; in which, most of them are redundant. The data with weakly influence in prediction increase the calculation load and time. In addition, the high collinearity among whole wavebands has negative influence on accuracy and robustness of models. Therefore, removing these redundant variables has a significant effect on improving data processing and accuracy of predictive models (Dai, Cheng, Sun, & Zeng, 2015). In the current study, a combination of UVE and backward regressing (BR) was employed to select the most informative wavelengths. UVE is a wavelength selection method based on regression coefficients of PLSR model by setting a threshold which eliminates variables with no or little information. However, the wavelengths selected by UVE is still large (Wu, Wu, Cai, Huang, & He, 2009). Therefore, in current study, BR was carried out after UVE to decrease data volume and select the informative but without collinearity variables. BR method eliminates variables one by one, according to a specific exit threshold (Gauchi & Chagnon, 2001). The procedure of UVE and BR was conducted in MATLAB R2016a software.

### 2.9 Model evaluation

The full spectrum and the spectral data selected by the combination of UVE and BR (UB) method were considered as input of linear and nonlinear models, and the predictive capability of these four models was compared. The assessment factors include the adjusted determination coefficient ($R_{\text{C}(\text{adj})}^{2}$, $R_{\text{CV}(\text{adj})}^{2}$, and $R_{\text{P}(\text{adj})}^{2}$), the root‐mean‐square error of them (RMSEC, RMSECV, and RMSEP), and bias. $R_{\left(\text{adj}\right)}^{2}$s and RMSEs were calculated by following equations:

(1) $$R^{2}=1-\frac{\sum {\left(y_{i,\text{pred}}-y_{i,\text{act}}\right)}^{2}}{\sum {\left(y_{i,\text{pred}}-\overset{¯}{y}\right)}^{2}},$$

(2) $$R_{\text{Adj}}^{2}=1-\left(1-R^{2}\right)\frac{\left(m-1\right)}{m-p-1},$$

(3) $$\text{RMSE}=\sqrt{\frac{1}{m}\sum_{i\;=\;1}^{m}{\left(y_{i,\text{pred}}-y_{i,\text{act}}\right)}^{2}},$$

*R* ^2^, coefficients of determination; $R_{\left(\text{adj}\right)}^{2}$, adjusted determination coefficient; *y_i_*_,pred_, the predicted value of TBARS; *y_i_*_,act_, the actual value of TBARS; *m*, the number of samples and *p*, the number of wavelengths.

Generally, a suitable prediction model should have higher values of $R_{\text{C}}^{2}$, $R_{\text{CV}}^{2}$, and $R_{\text{P}}^{2}$ (*R* ^2^ < 0.82: poor model; 0.82 ≤ *R* ^2^ ≤ 0.9: good model; and *R* ^2^ > 0.9: excellent model) and lower values of RMSEs as well as a small difference between them (Cheng & Sun, 2015c).

## 3 RESULTS AND DISCUSSION

### 3.1 Lipid oxidation of fish fillets

**Figure 1:**
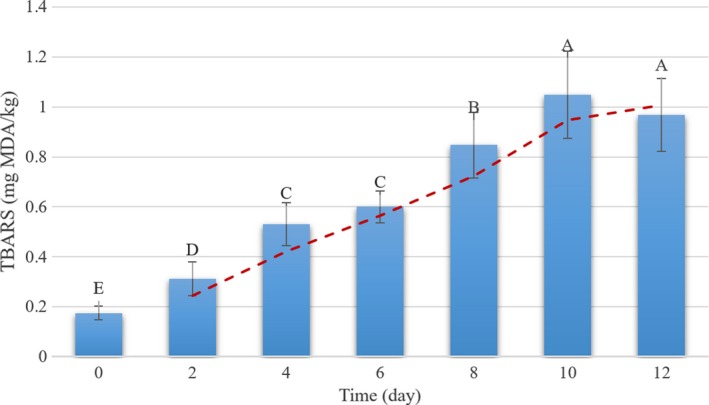
Reference measurement values of thiobarbituric acid‐reactive substances (TBARS) in rainbow trout fillets during cold storage time

The range of TBARS content was 0.134–1.36 mg MDA/kg, with mean and standard deviation of 0.646 and 0.325 MDA/kg (Table 1). The broad ranges of TBARS values showed that the samples can be represented by the actual possible range of TBARS content. It was helpful to establish the robust predictive models (Yang, Sun, & Cheng, 2017). Moreover, the status of lipid oxidation, regarding TBARS content, in fish fillets is presented in Figure 1. The TBARS content of samples significantly increased with storage time from 0 to 10 days of storage (*p* < 0.05), while afterward, the decreasing trend was observed in lipid oxidation status of samples. Similar behavior of TBARS values was also observed by previous researchers on rainbow trout fish fillet (Jouki, Yazdi, Mortazavi, Koocheki, & Khazaei, 2014; Rezaei & Hosseini, 2008). The decrease in TBARS values could be related to the reaction of MDA with amino acids, carbohydrates, and other different fish composition (Tsoukalas, Katsanidis, Marantidou, & Bloukas, 2011). Overall, time/temperature histories are the most important factors that can affect the freshness, safety, and quality of the food products (Abbas, Mohamed, Jamilah, & Ebrahimian, 2008). The values of the Pearson correlation coefficient revealed a good relationship between time and TBARS content (*R* = 0.916; *p* < 0.0001). This shows that TBARS can be a suitable indicator to evaluate freshness of rainbow trout fish samples.

**Table 1:** Descriptive statistics for thiobarbituric acid‐reactive substances value for rainbow trout samples measured by the conventional methods during 12 days of storage at 4 ± 2°C

Set	*N*	Mean	*SD*	Max	Min	Range
Calibration	98	0.651	0.332	1.36	0.134	1.23
Prediction	49	0.636	0.314	1.23	0.138	1.092
All	147	0.646	0.325	1.36	0.134	1.23

Abbreviation: *SD*, standard division.

### 3.2 Spectral features analysis

**Figure 2:**
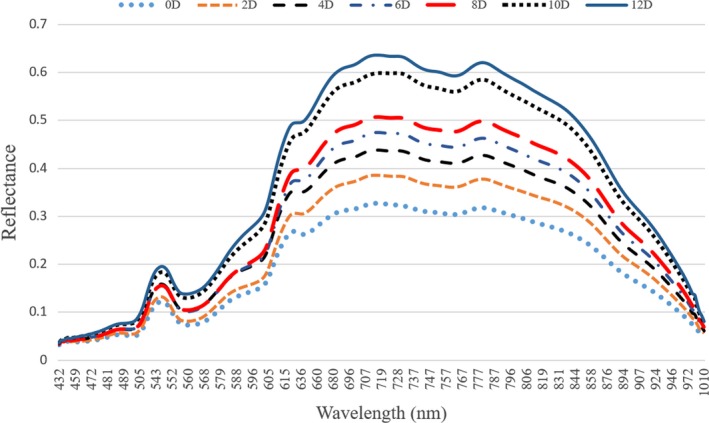
The mean spectral features of rainbow trout fillets at different cold storage days

The mean spectra of the rainbow trout images during different cold storage days within the spectral range of 430–1,010 nm are presented in Figure 2. The spectral reflectance curves of samples showed almost similar trend. But, it was obvious that the magnitudes of the curves tend to increase progressively with storage time, which mainly attributed to physical variations and decomposed chemical compound of fish muscle inducing by microbial and oxidative spoilage and enzyme activity over time (Xiong, Sun, Pu, et al., 2015; Xu et al., 2016). Therefore, the curves with lower reflectance were observers for fresh samples with low TBARS values and the higher ones were found in the spoiled samples (days 10 and 12).

**Figure 3:**
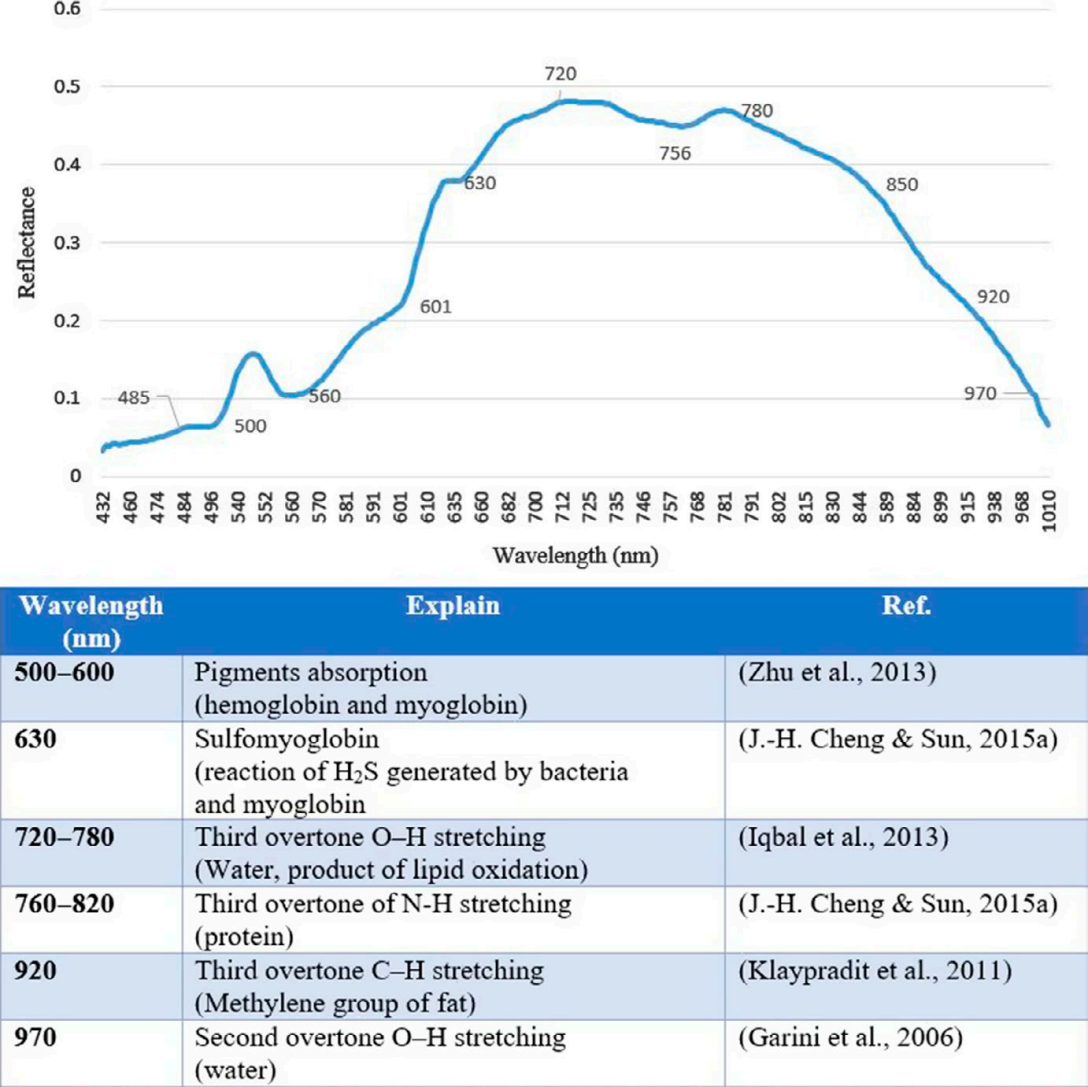
Important peak absorbtion of the rainbow trout fillets

Overall, absorption bands around visible spectral range (430–700 nm) can be explained by the absorption of pigments in fish fillets and absorption bands positioned in the NIR region (700–1,000 nm) are due to the overtone and vibrations of the molecular chemical bonds, such as C–O, O–H, C–H, S–H, and N–H (Cheng, Sun, Pu, Wang, et al., 2015; Cheng, Sun, Pu, & Zhu, 2015; Garini, Young, & McNamara, 2006; Iqbal, Sun, & Allen, 2013; Klaypradit, Kerdpiboon, & Singh, 2011; Sun et al., 2001; Xiong, Sun, Pu, et al., 2015; Zhu, Zhang, He, Liu, & Sun, 2013). Figure 3 shows the several obvious absorption bands and some possible explanations for them.

### 3.3 Prediction of TBARS content based on full wavelengths

In general, when a large number of samples are analyzed, the interpretation changes of the spectral profiles and establishing the relation between these changes and spoilage indicators are complicated. On the other hands, the spectral data can contain the hidden information relating to freshness indicator (such as TBARS value). Therefore, the use of chemometric analysis make it feasible to mine detailed information and extract the useful data from spectral plot of samples for prediction of TBARS value (Cheng & Sun, 2015c; Khoshnoudi‐Nia et al., 2018).

In current study, first one linear (PLSR) and one nonlinear (LS‐SVM) calibration models based on whole wavelengths (281 variables) were established to predict TBARS value. The performance of both PLSR and LS‐SVM models in the calibration, cross‐validation, and prediction sets were summarized in Table 2. From a comparison of the linear and nonlinear models, it is evident that the LS‐SVM model provided superior results than PLSR model ($R_{\text{P(adj)}}^{2}$ = 0.748, RMSEP = 0.155 mg MDA/kg vs $R_{\text{P(adj)}}^{2}$ = 0.829, RMSEP = 0.128 mg MDA/kg). Therefore, prediction power of LS‐SVM for evaluating TBARS was good ($R_{\text{P}}^{2}$ > 0.82). Unlike spectroscopy technique, data of HSI system may be suffered by stray light which are nonlinear factors (Zhang et al., 2018). Moreover, the oxidative spoilage process in food is a complex phenomenon and various interactions between fish composition and oxidation products during storage time causing several variations in TBARS values. Thus, relationship between TBARS values and the hypercube data can be tended to the nonlinearity. Similarly, several previous studies reported that the superior of nonlinear regression models over linear models for predicting various food quality and safety indicators (Cheng & Sun, 2015b; Cheng, Sun, Pu, & Zhu, 2015; Cheng et al., 2017; Lee, Kim, Lee, & Cho, 2018).

**Table 2:** Model performance for prediction of TBARS values in rainbow trout fillet during cold storage by hyperspectral imaging method

Model	*n*	LVs	TBARS (mg MDA/kg)					
			Calibration		Cross‐validation		Prediction	
			$R_{\text{C}(\text{adj})}^{2}$	RSMEC	$R_{\text{CV}(\text{adj})}^{2}$	RSMECV	$R_{\text{P}(\text{adj})}^{2}$	RSMEP
PLSR	281	10	0.787	0.152	0.743	0.167	0.748	0.155
LS‐SVM	281	–	0.852	0.130	0.834	0.138	0.829	0.128
UB‐PLSR	9	7	0.837	0.133	0.781	0.157	0.752	0.152
UB‐LS‐SVM	9	–	0.854	0.129	0.836	0.137	0.831	0.125
UB‐MLR	9	–	0.837	0.141	0.792	0.151	0.767	0.158
UB‐BP‐ANN	9	–	0.848	0.130	0.821	0.144	0.805	0.131

Abbreviations: BP‐ANN, back‐propagation artificial neural network; LS‐SVM, least squares support vector machine; LV, latent variable; MLR, multiple linear regression; PLSR, partial least squares regression; $R_{\text{C}(\text{adj})}^{2}$, adjusted determination coefficient of calibration; $R_{\text{CV}(\text{adj})}^{2}$, adjusted determination coefficient of cross‐validation; $R_{\text{P}(\text{adj})}^{2}$, adjusted determination coefficient of prediction; RMSEC, root‐mean‐square errors estimated by calibration; RMSECV, root‐mean‐square errors estimated by cross‐validation; RMSEP, root‐mean‐square errors estimated by prediction; TBARS, thiobarbituric acid‐reactive substances; UB, a combination of uninformative variable elimination and backward regression.

### 3.4 Selection of optimal wavelengths

**Figure 4:**
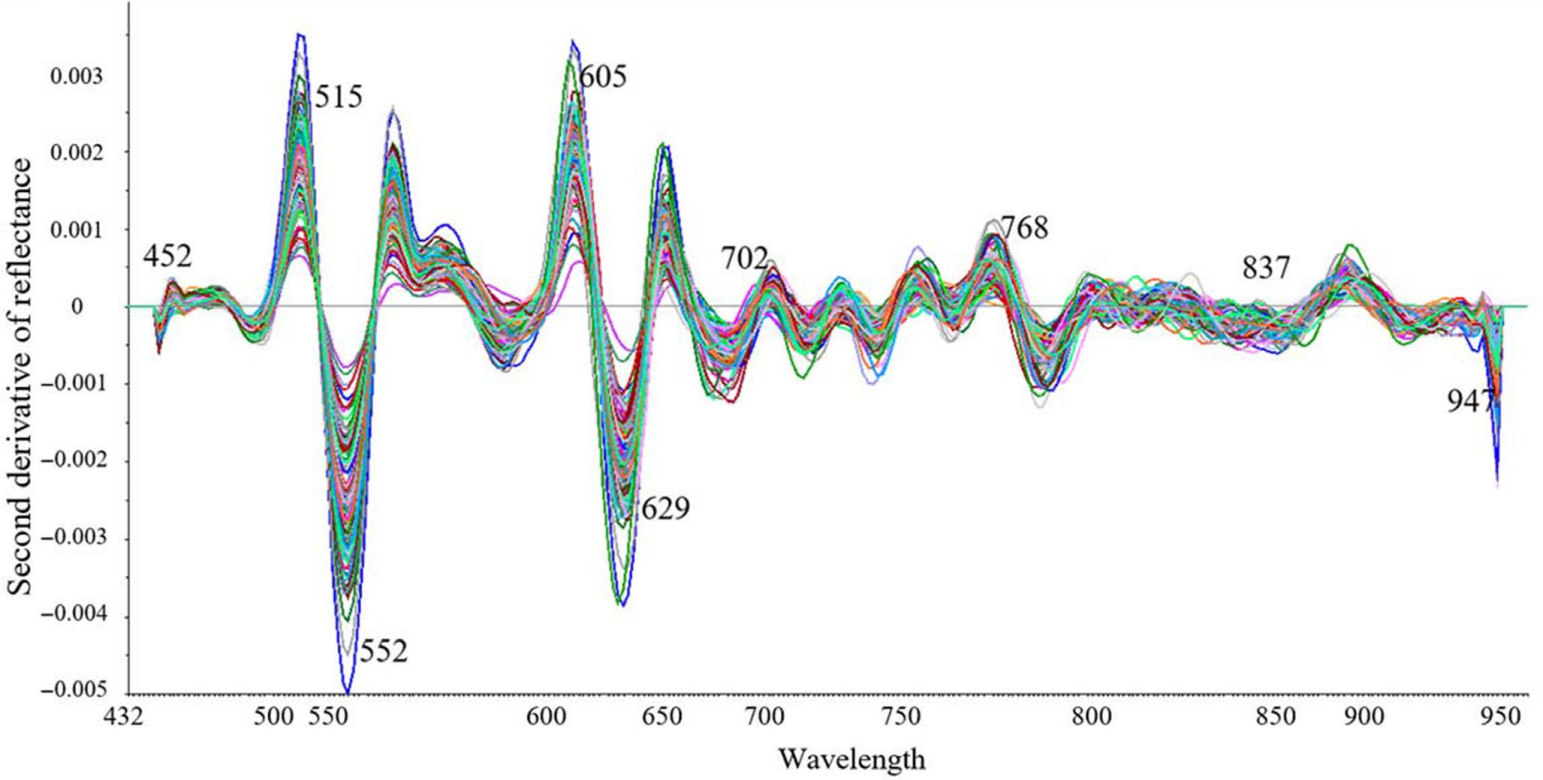
Exhibition of optimal wavebands on second derivative spectra plot

Although LS‐SVM model based on the full wavelengths could obtain a good performance for prediction of the TBARS values in rainbow trout fish fillets, it was still difficult to develop a real‐time detection system based on 281 variables. Therefore, to minimize computation time, hardware requirements, and dimensionality of the data for calibration, elimination of redundant wavebands from the spectral dataset seems an important step (Liu, Sun, & Zeng, 2014). In this study, a combination of UVE and BR methods was used to select the most effective wavelength variables carrying the valuable information related to the TBARS value of rainbow trout fillets from the whole spectral region. As the result, nine wavelength variables (452, 515, 552, 605, 629, 702, 768, 837, and 947 nm) were chosen as the optimal wavelengths. Figure 4 shows these wavebands on the second derivative spectra plot. Second derivative analysis was effective in making obvious some hidden information and peak absorption at original spectra plot (Cheng, Sun, Pu, & Wei, 2018). These wavelengths almost covered the whole spectral range, and they were mainly located on the visible region (such as 452, 515, 552, 605, 629, and 702 nm). The possible reasons were allied to the variations of color during storage which can be due to the chemical, microbial, and enzymatic reaction in fish fillets correlated with changes of TBARS content (Cheng, Sun, Pu, & Zhu, 2015; Hu et al., 2012). Among the peaks, those of 452, 515, 552, and 605 nm correspond to pigments that decompose during storage and show a lower reflectance (Khoshnoudi‐Nia & Moosavi‐Nasab, 2019), and that around 630 nm corresponded to sulfo‐myoglobin which increase during storage time and affect the shape of peak around this waveband. Moreover, those peaks about 750 and 950 nm reflected the third and second overtone O–H of water, respectively. The peak around 850 nm was usually assigned to the stretching overtones of C–H band of MDA and N–H bonds of protein and other organic compositions (Howard & Kjaergaard, 2006). These assignments showed that UVE–BR method was effective in wavelength selecting.

### 3.5 Prediction of TBARS content based on optimal wavelengths

After selecting optimal wavebands by UVE–BR method, these selection variables were applied to establish simplified prediction model. The performance of simplified linear (UB‐PLSR and UB‐MLR) and nonlinear (UB‐LS‐SVM and UB‐BP‐ANN) models for evaluation of TBARS values is summarized in Table 2. By comparing the models built using the whole dataset, the accuracy of the simplified models obtained comparable or even better results suggesting that the combination of UVE and BR method was successful in selection variable procedure. The simplified models have lower dimensionality which could be beneficial to develop a real‐time detection system (Xiong, Sun, Pu, et al., 2015). Since, in similar condition, the linear models were preferred over nonlinear models, for online and real‐time evaluation of the freshness indicator, the comparison between linear and nonlinear model is important to find the optimum model for rapid evaluation of quality properties (Wu & Sun, 2013c). As shown in Table 2, the prediction capability of nonlinear models was better than those of linear regression models. Among simplified models, only the UB‐LS‐SVM model yielded acceptable results with $R_{\text{P}}^{2}$ of 0.831 and RMSEP of 0.125 mg MDA/kg (0.82 ≤ *R* ^2^ ≤ 0.9). The results of current study were weaker than those reported by Cheng, Sun, Pu, Wang, et al. (2015) who selected the optimal wavelength by regression coefficient (RC) and revealed that RC‐MLR ($R_{\text{P}}^{2}$ = 0.839 and RMSEP = 0.115) had a better performance than RC‐PLSR ($R_{\text{P}}^{2}$ = 0.832 and RMSEP = 0.117) for prediction of TBA value in grass carp fillet (Cheng, Sun, Pu, Wang, et al., 2015). However, the results acquired by Xiong, Sun, Xie, et al. (2015) for prediction of TBARS value in chicken meat using SPA‐PLSR were poor with $R_{\text{P}}^{2}$ of 0.641 and RMSEP of 0.157 (Xiong, Sun, Pu, et al., 2015). Therefore, besides type of calibration model and selection variable method, type and number of samples can also be effective on quality prediction performance.

During storage of fish, MDA was involved in various interactions with different compositions of sample (such as amino acids, proteins, and glucose). Therefore, this factor subjected to several fluctuations (Fernández, Pérez‐Álvarez, & Fernández‐López, 1997; Rezaei & Hosseini, 2008). This makes the prediction of TBARS values more complex in rainbow trout.

## 4 CONCLUSIONS

A HSI technique system (432–1,010 nm) coupled with linear and nonlinear chemometric analysis was applied to evaluate TBARS content, as an important freshness factor, in rainbow trout fish fillet. To minimize computation load and make suitable model for real‐time TBARS evaluation, a combination of UVE and BR method was used to select the most effective wavelength variables and nine wavelength variables (452, 515, 552, 605, 629, 702, 768, 837, and 947 nm) were selected. Four simplified models based on MLR, PLSR, BP‐ANN, and LS‐SVM model were established and compared to find the optimal TBARS value prediction model. The results showed that the nonlinear model has superior over linear model. However, only LS‐SVM model yielded acceptable results with $R_{\text{P}}^{2}$ of 0.831 and RMSEP of 0.125 mg MDA/kg. Although, the results of this study demonstrated the potential of HSI system, as a replacement for traditional methods, for direct and noninvasive measurement of TBARS values in rainbow trout fillets. But, the establishing of lipid‐oxidation prediction model in rainbow trout fish was complicated, due to the fluctuations of TBARS values during storage. Therefore, further researches are needed to improve the prediction results and applicability of HSI technique for prediction of TBARS value in rainbow trout fish and the predictive power and applicability of the models by investigating and developing the new chemometric algorithms (such as deep learning models). To acquire the better evaluation of freshness of fish samples, the TBARS value can be determined simultaneously with the other freshness indicator.

## CONFLICT OF INTEREST

The authors declare that they have no conflict of interest.

## ETHICAL APPROVAL

This article does not contain any studies with human participants or animals performed by any of the authors.
